# Cohort Profile: The Hepatitis C Virus (HCV) Research UK Clinical Database and Biobank

**DOI:** 10.1093/ije/dyw362

**Published:** 2017-02-27

**Authors:** J McLauchlan, H Innes, J F Dillon, G Foster, E Holtham, S McDonald, B Wilkes, S J Hutchinson, W L Irving, on behalf of the HCV Research UK Steering Committee, HCV Research UK Steering Committee, K Agarwal, M Aldersley, A Ala, G Alexander, R Aspinall, S Barclay, E Barnes, S Bansal, M Bassendine, J Benselin, A Brown, J Butterworth, C Ch’ng, G Cooke, L Corless, M Cramp, S Datta, S Davison, J Dillon, D Forton, G Foster, M Foxton, A Fraser, W Gelson, A Gera, A M Geretti, D Goldberg, D Gorard, F Gordon, C Gore, H Harris, P Hayes, M Heydtmann, A Higham, E Holtham, Hubscher, S Hutchinson, W L Irving, N Jenkins, D Kelly, N Kennedy, S Khakoo, J Knowles, A Langford, A Lawson, C Leen, S Loganathan, S E McDonald, J McLauchlan, S McPherson, P Mills, S Moreea, D Mutimer, E Nastouli, K Neal, A Patel, M Priest, M Prince, P Quinlan, Y Reddy, P Richardson, W Rosenberg, S Ryder, P Shields, C Shorrock, S Singhal, A Sreedharan, R Srirajaskanthan, B Stone, M Thursz, G Tudor-Williams, A Ustianowski, S Verma, B Wilkes, M Wiselka

**Affiliations:** 1 MRC-University of Glasgow Centre for Virus Research, Glasgow, UK; 2 School of Health and Life Sciences, Glasgow Caledonian University, Glasgow, UK; 3 Blood Borne Viruses and STIs Division, Health Protection Scotland, Glasgow, UK; 4 Division of Molecular and Clinical Medicine; University of Dundee, Dundee, UK; 5 Institute of Cell and Molecular Science, Queen Mary University of London, London, UK; 6 National Institute for Health Research (NIHR) Digestive Diseases Biomedical Research Unit at Nottingham University Hospitals NHS Trust and the University of Nottingham, Nottingham, UK

## Why was the cohort set up?

Hepatitis C is a blood-borne virus that causes scarring and inflammation of the liver.^1^ The virus is also associated with extrahepatic disease, including non Hodgkins lymphoma,^2^ renal disorders^3^ and, probably, cardiovascular disease.^4^ Chronic hepatitis C virus (HCV) infection-referring to persistent carriage of the virus is a global problem affecting in excess of 140 million individuals^5^ and causing half a million deaths every year from liver disease.^6^

HCV Research UK is a consortium of leading stakeholders in the UK, including all the major adult and paediatric liver centres. The remit of HCV Research UK is to address critical gaps in our understanding of this virus. These gaps exist particularly in relation to: (i) the clinical course of HCV-related disease; (ii) the optimal clinical management of the virus (including the effectiveness and long-term impact of antiviral therapies); and (iii) the viral and host genetic factors influencing prognosis.

The HCV Research UK clinical database and biobank was set up in 2012 to advance our knowledge in these areas. It includes in excess of 10 000 patients from across the UK, who have attended a specialist HCV clinic for care/management of their HCV infection. Extensive epidemiological and clinical data have been collected for each participant, both at baseline and longitudinally thereafter, but the unique strength of this cohort is that each participant has further submitted a blood specimen for secure storage in a biorepository (‘biobank’). These biological samples can be used for viral and host genotyping as well as other appropriate research analyses. It is envisaged that integrating biological, epidemiological and clinical data in this way will encourage a more multidisciplinary approach to HCV research.

The clinical database is managed at the University of Nottingham, and the biobank is sited at the Centre for Virus Research (CVR) along with a satellite biobank at the University of Birmingham. In this profile report, we provide a broad overview of this cohort, including a detailed description of the participants, a summary of the data collected and an outline of its successes so far.

## Who is in the cohort?

**Figure 1:**
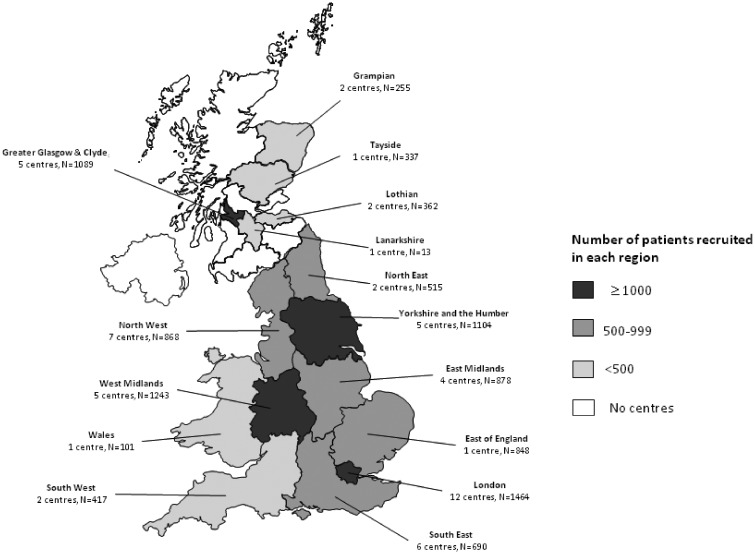
Breakdown of HCV Research UK cohort sites (N = 56) and participants (N = 10 184) by geographic region.

In total, 10 184 patients with past or current chronic infection were enrolled into the cohort through attendance at one of 56 specialist UK HCV clinics between March 2012 and July 2015. Figure 1 illustrates the geographical distribution of these specialist clinics and the overall contribution of each UK region to the cohort. Almost all of the highly populated parts of the UK are represented in the cohort, with most participants recruited from Yorkshire, London, the West Midlands and Glasgow. Exclusion criteria for recruitment included an inability to obtain informed consent or being detained in prison at the time of clinic appointment. Table 1 shows that the vast majority (82.6%) of participants recruited were existing patients of the specialist HCV clinic and had been in attendance for a median of 3.7 years [interquartile range (IQR] 1.3–8.1). Otherwise, 15.2% of the final cohort were recruited as new referrals to the HCV clinic, and thus their date of cohort enrolment coincides with their first attendance date at the specialist centre. The remaining 2.3% of the cohort were patients who had previously attended a specialist HCV clinic but had been discharged following clearance of their infection through antiviral treatment (known as a sustained virological response or SVR). These patients who had achieved an SVR were invited back to participate in this cohort and would not have attended the clinic otherwise.

**Table 1:** Break down of HCV Research UK cohort (N = 10 184) according to recruitment approach and time between first appointment at specialist clinic and enrolment

Recruitment approach	N (%)*	Years from first clinic attendance to enrolment, median (IQR)
New attender	1476 (15.2)	0 (0.0–0.1)
Existing attenders	8039 (82.6)	3.7 (1.3–8.1)
Discharged sustained viral responder	219 (2.3)	8.1 (3.2–12.6)
Unknown	450 (–)	1.7 (1.7–4.3)

*Percentages exclude individuals with unknown recruitment approach.

Table 2 describes some of the key characteristics of the cohort. The mean age at baseline is 48.5 years, and although most (58%) participants were aged between 45 and 64 years, the cohort also includes a subgroup of 85 children (age < 16 years). Overall, participants are typically male (70.9%), of White ethnicity (83.4%) and acquired their infection through injecting drug use (53.9%). The average duration of infection at enrolment is in excess of 20 years (23.4 years). At enrolment, the majority had current chronic infection, although an important subset (13.4%) had past chronic infection which they had previously cleared through treatment (i.e. through attaining an ‘SVR’). In terms of the clinical consequences of chronic infection, almost a quarter of the cohort have been diagnosed with liver cirrhosis, in either compensated (18.2%) or decompensated form (5.2%), and 2% of participants had liver cancer. Of the specialist HCV clinics participating in HCV Research UK (see Figure 1), around 50% of their total HCV clinic population were recruited into our final cohort (based on available data from 29 clinics). To assess how representative this cohort is of all patients attending specialist care, we compared the broad demographics of our final cohort with those of all patients attending clinical services in Scotland (the only region in the UK where a national clinical database of HCV attendees exists). Table 3 shows that HCV Research UK participants recruited from Scottish centres are broadly comparable to all Scottish attendees in terms of age, gender, and ethnicity. The only notable difference was that for the HCV Research UK cohort, patients were less likely to be assigned an unknown route of infection (10.4% in Scottish HCV Research UK participants versus 25.2% for all Scottish attendees). This may reflect a more extensive probing for risk factor data in HCV Research UK than that carried out routinely in the Scottish national database.

**Table 2:** Description of the final cohort at the time of enrolment (N = 10 184)

Characteristic				N (%)
Demographic factors	Age in years	<16		85 (0.8)
		16–44		3423 (33.7)
		45–64		5934 (58.4)
		65+		718 (7.1)
		Missing		24 (0.2)
		mean age (SD)		48.5 (11.7)
	Gender	Female		2950 (29.0)
		Male		7190 (70.6)
		Other/missing		44 (0.4)
	Ethnic group	White		8496 (83.4)
		Asian/Asian British		1004 (9.9)
		Black/African/Caribbean/Black British		223 (2.2)
		Mixed/multiple ethnic groups		103 (1.0)
		Other		256 (2.5)
		Unknown		102 (1.0)
	Country of birth	UK		7391 (72.6)
		non-UK		2591 (25.4)
		NK/missing		202 (2.0)
Hepatitis C infection factors	Route of infection/ risk group	Injecting drug use		5290 (51.9)
		Blood transfusion/blood products		1158 (11.8)
		Born abroad		1006 (9.9)
		Sexual transmission		335 (3.3)
		Perinatal transmission		98 (1.0)
		Other		1074 (10.6)
		No known risk factor		657 (6.5)
		Missing		566 (5.6)
	Estimated duration of infection*	<20 yrs		2840 (27.9)
		20yrs+		4214 (41.4)
		unknown		3130 (30.7)
		Median duration, years (IQR)		24 (13–32)
	Genotype	1		4770 (46.8)
		2		446 (4.4)
		3		3646 (35.8)
		4,5,6		338 (3.3)
		Mixed genotype		14 (0.1)
		Missing/NK		970 (9.5)
Clinical factors	Antiviral treatment history	Never treated		4002 (39.3)
		Currently being treated		1511 (14.8)
		Previously treated and cleared infection (i.e. SVR)		1366 (13.4)
		Previously failed treatment (i.e. non-SVR)		2947 (28.9)
		Missing/NK		358 (3.5)
	Liver-related consequences of hepatitis C infection	Diagnosed with liver cirrhosis	No	7803 (76.6)
			Yes - compensated cirrhosis	1851 (18.2)
			Yes - decompensated cirrhosis	530 (5.2)
		Diagnosed with liver cancer	No	9981 (98.0)
			Yes	203 (2.0)
		Prior liver transplantation	No	9674 (95.0)
			Yes	495 (4.9)
			Unknown	15 (0.2)
**All patients**				**10184**

*For individuals who acquired their infection via injecting drug use, the duration of infection was estimated by subtracting the year of enrolment into HCV Research UK from the year of first injection. We then subtracted a further 3 years from this, which relates to the average time from commencing injecting drug use to acquiring HCV infection (Hagan et al, Epidemiology. 2004;15(5):543–9).

**Table 3:** Characteristics of patients enrolled into the HCV Research UK cohort compared to all patients attending hepatitis C specialist services in Scotland

Characteristic		HCV Research UK		Scottish National HCV clinical database
		All centres (N=10,184)	Scottish Centres (N=1785)	All HCV patients attending Scottish specialist services, April 2012–April 2015, (N=9787)
		N(%)	N(%)	N(%)
Gender	Female	2950 (29.0)	496 (27.8)	2894 (29.6)
	Male/other/missing*	7234 (71.0)	1289 (72.2)	6893 (70.4)
Age in years	<16	85 (0.8)	0 (0.0)	18 (0.2)
	16–44	3423 (33.7)	840 (47.1)	5671 (57.9)
	45–64	5934 (58.4)	876 (49.1)	3815 (39.0)
	65+	718 (7.1)	63 (3.5)	283 (2.9)
	Missing	24 (0.2)	6 (0.3)	0 (0.0)
	mean age (SD)	48.5 (11.7)	45.2 (10.2)	43.4 (14.3)
Ethnic group	White	8496 (83.4)	1697 (95.1)	9327 (95.3)
	Asian/Asian British	1004 (9.9)	60 (3.4)	294 (3.0)
	Black/African/Caribbean/Black British	223 (2.2)	7 (0.4)	29 (0.3)
	Any other	359 (3.5)	17 (1.0)	52 (0.5)
	unknown	102 (1.0)	4 (0.2)	85 (0.9)
Route of infection/risk group	Injecting drug use	5290 (51.9)	1203 (67.4)	6031 (61.6)
	Blood transfusion/blood products	1158 (11.4)	168 (9.4)	530 (5.4)
	Sexual transmission	335 (3.3)	60 (3.4)	215 (2.2)
	Other**	2178 (21.4)	168 (9.4)	548 (5.6)
	unknown/ no known risk factor	1223 (12.0)	186 (10.4)	2463 (25.2)

*Males collapsed with other/missing category to minimise risk of disclosure and to help maintain confidentiality.

**The “born abroad” risk group for HCV Research UK participants has been collapsed into the “other” category, because no equivalent risk group to “born abroad” exists in the Scottish clinical database. “Perinatal transmission group has also been collapsed into “other” to minimise the risk of disclosure and to help maintain confidentiality.

Within the HCV Research UK cohort described above, are two subgroups that have been subject to more detailed investigation and scrutiny. The first subgroup comprises individuals who took part in the NHS England Expanded Access Programme (EAP) that was launched in June 2014 (*N* = 806) [7]. The purpose of this programme was to provide patients at greatest need (i.e. mainly those with advanced liver disease) with early access to the new but highly costly all-oral antiviral regimens. The second subgroup comprises patients who participated in the STOP-HCV study (*N* = ∼4000) [www.stop-hcv.ox.ac.uk]. STOP-HCV is a £5.2 million MRC-sponsored study that aims to use epidemiological, clinical and genetic patient information to establish the most effective and cost-effective treatments for patients with HCV. Within this STOP-HCV subgroup is a further notable subset of patients who are taking part in the STOP-HCV cirrhosis sub-study (*N* = 1200). This cirrhosis sub-study is a 5-year prospective investigation into the determinants of liver disease progression in patients with compensated cirrhosis.

Further to the 10 184 individuals with past/current chronic infection, HCV Research UK also recruited 275individuals with ‘spontaneously resolved’ infection. Spontaneous resolution refers to natural clearance of HCV without treatment, and occurs in a quarter of all persons infected (usually within 6 months of the initial infection event).^8^ Because the number of spontaneous resolvers recruited was small and because spontaneous resolvers are not ordinarily referred to specialist clinics for care/management of HCV infection, these participants were treated as distinct and not included in the data tables of this profile.

Finally, there is ongoing recruitment of 1200 patients receiving therapy containing second-generation (i.e. not telaprevir or boceprevir) direct-acting antiviral agents in routine clinical practice [direct-acting antivirals (DAA) study].

## What has been measured and collected?

HCV Research UK collects baseline clinical and epidemiological data through a standardized questionnaire that is completed for each participant at enrolment. Follow-up data are collected mainly through assessment of patient medical notes. Table 4a provides a broad overview of the clinical and epidemiological information collected on each participant. In brief, the data collected relate to: (i) demographic factors; (ii) acquisition of HCV; (iii) health risk behaviours; (iv) physical characteristics of participant; (v) hepatitis C virology; (vi) comorbid health conditions; (vii) co-medications; (viii) liver disease status; (ix) HCV antiviral treatment episodes; (x) routine laboratory tests; and (xi) vital status. All data are entered directly at the recruitment site into the database by trained clinical staff. The database itself is hosted by an IT company (Illuminaries Ltd) on a server behind the NHS firewall. At the time of enrolment, each patient is assigned a unique study number that is used to link the above clinical/epidemiological data with biological samples. These biological samples take the form of blood specimens that are drawn at enrolment, and sent by overnight courier at ambient temperature to the central biorepository at the CVR. On arrival, samples (serum and plasma) are processed, aliquoted and placed in long-term storage at -80°C. The buffy coat layer from EDTA-treated blood is also collected and stored for preparation of DNA. Full-length next-generation sequencing of viral RNA is being carried out on ∼ 2000 individuals. This includes the 806 participants with advanced liver cirrhosis, who were treated with all-oral DAAs in the NHS England EAP,^7^ and also patients in the STOP-HCV cirrhosis study. Host genotyping has been performed on ∼ 4000 participants and has focused on patients with genotype 3 infection and/or cirrhosis (see Table 4b). Host genotyping was carried out using the Affymetrix UK biobank microarray, which generates data on ∼ 800 000 single nucleotide polymorphisms, including polymorphisms in the interleukin 28b gene (on chromosome 19) that have previously been shown to influence hepatitis C treatment response^9^ and liver disease progression.^10^

**Table 4a:** HCV Research UK cohort: summary of epidemiological and clinical data collected for all participants

Category	Major variables captured	Time point	
		Enrolment	Follow-up (at least bienially following enrolment)
1: Socio-demographic factors	gender; age; country of birth; ethnicity; recruitment approach; first clinic attendance date	Y	N/A
2: HCV acquisition	route of infection; estimated year of infection, date of HCV diagnosis	Y	N/A
3: Health risk behaviours	data on: alcohol use; drug use; tobacco smoking; and cannabis smoking	Y	Y
4: Physical characteristics	Height, weight, BMI	Y	Y
5: HCV virology	HCV genotype; HCV genotype subtype; viral RNA positivity status; quantitative viral load	Y	Y
6: Comorbid health conditions	presence of: renal failure; diabetes; cancer; depression; HIV co-infection; coagulation bleeding disorder; cryoglobulinaemia	Y	Y
7: Co-medications taken	use of: anti-diabetics, anti-depressants, anti-retrovirals, hypnotics, immunosupressive drugs; opiate substitution therapy; steroids; statins	Y	Y
8: Liver disease status	data on: liver transplantation; liver biopsies performed; diagnosis of cirrhosis; portal hypertension; diagnosis of decompensated cirrhosis; diagnosis of liver cancer; fibroscan scores.	Y	Y
9: Hepatitis C antiviral treatment	data on HCV antiviral treatment episodes including: outcome (i.e. viral clearance), treatment regimen;duration of treatment and side effects.	Y	Y
10: Routine laboratory data	liver function tests; full blood counts; lipid profile tests; blood sugar tests; and renal function tests	Y	N
11. Vital status	data on date and cause of death	N/A	Y

Y= Yes; N=No; N/A = not applicable.

**Table 4b:** Overview of enhanced data collection for strategic subgroups of the HCV Research UK cohort

Subgroup	N (% of full cohort)	Additional data collected
Participants taking part in the NHS England early access programme	806 (7.9%)	Data on : 1) routine laboratory results collected annually; 2) prospective health resource utilisation data*; 3) viral sequencing on baseline, during-therapy and post-therapy samples; 4) host genotyping for ∼800,000 single nucleotide polymorphisms
Genotype 3 infected participants taking part in STOP-HCV	∼2000 (19.6%)	Data on : 1)host genotyping for ∼800,000 single nucleotide polymorphisms
Participants in the STOP-HCV cirrhosis sub-study	∼1200 (11.8%)	Data on : 1) routine laboratory results collected annually; 2) viral sequencing on baseline samples; and 3) host genotyping for ∼800,000 single nucleotide polymorphisms

*Refers to data on all HCV-related clinic visits; treatment of adverse effects from antivirals; and general hospital admissions.

## How often have they been followed up?

Data on health risk behaviours, physical characteristics, HCV virology, comorbidities, co-medications, liver disease status, antiviral treatment episodes and mortality status are collected at least biennially for all participants in the cohort (see Table 4a). Enhanced follow-up data for key strategic subgroups of the cohort are also being captured (see Table 4b). This includes the collection of routine laboratory data for all individuals who took part in the NHS England EAP, and for individuals in the STOP-HCV cirrhosis sub-study. Prospective follow-up data on health care utilization encompassing information on HCV-related clinic visits, treatment of adverse effects from antivirals, and general hospital admissions are also being collected for those patients in the EAP. Such data on health care utilization are intended to inform estimates of treatment cost-effectiveness which is currently a critical topic within HCV research, given the high cost of new medicines.^11^ Finally, in time, prospective data collection for the bulk of the cohort on hospital admissions and cancer registries is planned through record linkage to UK nationwide health registries.

## What has it found? Key findings and publications

Although the merits of clearing HCV through antiviral treatment are well studied for patients with compensated (i.e. asymptomatic) liver disease, little is known about the benefits of treatment once advanced symptomatic disease has set in. This cohort has duly contributed one of the first studies to address this gap in the evidence base.^12^ The study examined the impact of antiviral treatment on a patient’s Model for End-Stage Liver Disease (MELD) score, which is a surrogate measure of liver function and ranges from a value of 6 (representing the lowest illness severity) up to a value of 40 (representing the highest illness severity). Among 467 patients with advanced liver disease, who received a 12-week course of antiviral treatment, the mean post-treatment MELD score was modestly lower than the mean pre-treatment MELD score (mean improvement of -0.85). In contrast, among 261 untreated patients followed up for a comparable time frame, the MELD score deteriorated (mean change of +0.75). This provides some initial evidence that antiviral treatment may be of value to patients with advanced disease albeit, going forward, it will be important to confirm this putative benefit against hard clinical endpoints such as all-cause mortality. Otherwise, there is a rich pipeline of research projects that are currently in progress. For example, this cohort underpins the aforementioned MRC-funded STOP-HCV study, which aims to inform how a ‘stratified medicine’ approach can be applied to the treatment of hepatitis C. Further, the Tissue and Data Access Committee (TDAC) of HCV Research UK have thus far approved more than 65 applications from academia and industry for access to data and samples. These applications cover a broad range of topics and scientific disciplines, including lipidomics, metabolomics and proteomics, in addition to proposals for more traditional epidemiological studies such as investigating the association between alcohol and disease progression. From our own initial review of the data, one particular topic that stands out as being worthy of in-depth research is the singular behaviour/comorbidity profile of this cohort. Table 5 shows that almost 40% have a history of heavy alcohol use, 6.6% have recently injected drugs, 54% are current smokers and 16% have previously attempted suicide; all these statistics are vastly out of step with the crude prevalence reported for the UK general population. Epidemiological studies to disentangle the impact on health of chronic infection itself versus these concomitant conditions would be of real value, as would the investigation of appropriate interventions to redress high levels of heavy alcohol use, drug use and mental health issues.

**Table 5:** Prevalence of selected health conditions/behaviours among participants of HCV Research UK at the time of enrolment

Health condition/behaviour	Prevalence among HCV Research UK participants, by gender	Crude prevalence in UK general population	Data source for crude population prevalence
Recent injecting drug use*	Male: 7.8%	<1%	UK report to European monitoring centre for drugs and drug addition, 2014^14^
	Female: 3.9%		
Past injecting drug use	Male: 62.4%	1%	
	Female: 46.6%		
Daily/occasional cannabis use	Male: 27.7%	7%	
	Female: 15.8%		
Current tobacco smoker	Male: 58.0%	19%	Health Survey England, 2014^15^
	Female: 45.3%		
Obesity (BMI≥30)	Male: 20.3%	26%	
	Female: 24.9%		
Underweight (BMI <18.5)	Male: 2.6%	2%	
	Female: 3.5%		
Diagnosed with diabetes	Male: 11.2%	6%	
	Female: 9.2%		
Previous suicide attempt	Male: 15.1%	6%	
	Female 20.2%		
History of sustained heavy alcohol use^†^	Male: 43.2%	2–12%^†^	
	Female: 23.6%		
Current excess alcohol use^‡^	Male: 8.7%	18%	
	Female: 7.7%		
Previous hospital admission for depression	Male: 9.1%	1%	Innes et al^16^
	Female: 12.4%		
HIV infected	Male: 6.0%	<1%	Public Health England, 2014^17^
	Female: 2.9%		

*Recent defined as within the past six months.

^†^Past sustained heavy alcohol use defined as drinking >50 units a week for at least 6 months. No directly comparable UK population statistic exists for this. As a lower bound, we have indicated 2% which refers to the prevalence of current alcohol consumption >50 units week taken from Health Survey England. As the upper bound we have indicated 12% which refers to the lower bound estimate added to the proportion of ex-drinkers in the UK population.

^‡^Current excess alcohol use refers to drinking >21 units in the last 7 days for males, and >14 units/wk for females.

## What are the main strengths and weaknesses?

This cohort has several notable strengths. Foremost, this is one of the largest HCV clinical database and biobank resources to have been established anywhere in the world. This resource will therefore open the door to new types of research study for instance, large-scale genome-wide association studies to identify host polymorphisms that influence liver fibrosis progression and development of hepatocellular carcinoma. Second, we have recruited across most of the geographical area of the UK, creating a network of 56 sites that includes the largest tertiary clinical centres that manage the care of HCV-infected individuals. As a consequence, it will be possible to map the viral strains and genotypes which currently circulate in the UK, aiding the identification of transmission networks and sub-groups with particular characteristics (e.g. those with cirrhosis). Such information could potentially inform prevention strategies and enable identification of any emerging novel resistant strains that arise as a consequence of large-scale use of all-oral, interferon (IFN)-free DAA therapeutic regimens. Finally, the cohort is sufficiently large to allow sub-studies on special-interest patient groups (e.g. paediatric patients, those with cirrhosis for disease progression, re-treatment in patients who previously did not respond to therapy and comparison of treatment response to new therapies across genotypes).

The following limitations should be highlighted. First, of all patients in attendance at the 56 HCV Research UK clinics (see Figure 1), only an estimated 50% were enrolled into the cohort. Given this moderate response rate, it is possible that participants differ from non-participants in epidemiologically and clinically important ways. Although the broad demographic comparability between the Scottish contingent of HCV Research UK and all patients attending an HCV clinic in Scotland (see Table 3) is reassuring it does not necessarily disprove the existence of selection bias. Furthermore, the cohort is unlikely to be representative of the total UK population that are living with HCV infection principally because most infected persons are not currently in contact with specialist HCV clinics, and indeed many have not even been diagnosed.^13^ These caveats should be borne in mind when interpreting data from this cohort. Second, almost all patients in the cohort had established or previous chronic infection at enrolment, and so there are no samples in the biorepository that span the acute phase of hepatitis C infection. Finally, for longitudinal studies, our collection of serial biological samples is somewhat limited. However, the 1200 STOP-HCV cirrhosis study patients will be sampled annually for 5 years, and the 806 patients included in the NHS EAP were serially sampled at enrolment, during treatment and after treatment.

## Can I get hold of the data? Where can I find out more?

The epidemiological data and biological samples are available to all researchers in both academia and the biopharmaceutical industry upon successful application to the HCV Research UK TDAC. Access requires submission of a formal application to the HCV Research UK TDAC using an online system available at [www.hcvresearchuk.org]. TDAC evaluates the merit of each proposal and also has the authority to grant ethical approval for any submitted studies. Decisions on submitted applications take up to 4 weeks, followed by completion of a Material Transfer Agreement or contract that sets out the terms for provision of the data and samples. One of the conditions for release of data and samples is that any results generated would be returned for inclusion in the HCV Research UK database following their publication. Additional information on accessing data and samples can be obtained from either of the co-chairs of HCV Research UK: Dr John McLauchlan [john.mclauchlan@glasgow.ac.uk] or Prof. Will Irving [will.irving@nottingham.ac.uk], and details regarding submission of applications are available from the Biobank Manager, Dr Sarah McDonald [sarah.mcdonald@glasgow.ac.uk].

**Profile in a nutshell**

- HCV Research UK was established to address critical gaps in our understanding of the hepatitis C virus. Participants are characterised in terms of epidemiological/clinical factors and have also submitted blood samples for storage in a bio-repository
- Includes in excess of 10,000 patients from across the UK who have attended a specialist HCV clinic for care/management of their HCV infection in 2012-2015
- Baseline data collected on: 1. Socio-demographic factors; 2. HCV acquisition; 3. Health risk behaviours; 4. Physical characteristics; 5. HCV virology; 6. Comorbid health conditions; 8. Co-medications; 9. HCV treatment; 10. Routine laboratory tests; and 11. Vital status
- Follow-up data collected biennially on selected clinical and epidemiological variables
- Data available to researchers upon successful application to the HCV Research UK Tissue and Data Access Committee. Applications available at: www.hcvresearchuk.org

## Funding

Funding to support this initiative was provided by the Medical Research Foundation, the independent charity of the UK Medical Research Council. HCV Research UK was established by a grant from the Medical Research Foundation (award no: C0365). The award was made to the MRC-University of Glasgow Centre for Virus Research (CVR) [www.gla.ac.uk/researchinstitutes/iii/cvr/].
